# Role of Integrin Alpha4 in Drug Resistance of Leukemia

**DOI:** 10.3389/fonc.2014.00099

**Published:** 2014-05-23

**Authors:** Stephanie Shishido, Halvard Bönig, Yong-Mi Kim

**Affiliations:** ^1^Division of Hematology and Oncology, Department of Pediatrics, Children’s Hospital Los Angeles, University of Southern California Keck School of Medicine, Los Angeles, CA, USA; ^2^Institute for Transfusion Medicine and Immunohematology, German Red Cross Blood Service Baden-Wuerttemberg-Hessen, Goethe University, Frankfurt, Germany

**Keywords:** integrin alpha4, CD49d, adhesion, drug resistance, acute lymphoblastic leukemia

## Abstract

Chemotherapeutic drug resistance in acute lymphoblastic leukemia (ALL) is a significant problem, resulting in poor responsiveness to first-line treatment or relapse after transient remission. Classical anti-leukemic drugs are non-specific cell cycle poisons; some more modern drugs target oncogenic pathways in leukemia cells, although in ALL these do not play a very significant role. By contrast, the molecular interactions between microenvironment and leukemia cells are often neglected in the design of novel therapies against drug resistant leukemia. It was shown however, that chemotherapy resistance is promoted in part through cell–cell contact of leukemia cells with bone marrow (BM) stromal cells, also called cell adhesion-mediated drug resistance (CAM-DR). Incomplete response to chemotherapy results in persistence of resistant clones with or without detectable minimal residual disease (MRD). Approaches for how to address CAM-DR and MRD remain elusive. Specifically, studies using anti-functional antibodies and genetic models have identified integrin alpha4 as a critical molecule regulating BM homing and active retention of normal and leukemic cells. Pre-clinical evidence has been provided that interference with alpha4-mediated adhesion of ALL cells can sensitize them to chemotherapy and thus facilitate eradication of ALL cells in an MRD setting. To this end, Andreeff and colleagues recently provided evidence of stroma-induced and alpha4-mediated nuclear factor-κB signaling in leukemia cells, disruption of which depletes leukemia cells of strong survival signals. We here review the available evidence supporting the targeting of alpha4 as a novel strategy for treatment of drug resistant leukemia.

## Introduction

Relapse of leukemia due to incomplete eradication of leukemia stem cells by conventional chemotherapy remains a problem in adult and childhood leukemia patients. The elimination of chemotherapy-refractory relapse-initiating acute lymphoblastic leukemia (ALL) cells thus remains a significant challenge and novel approaches for targeting residual leukemia cells are warranted. Even though chemotherapy kills the bulk of ALL cells, some can evade the toxicity of cytoreductive chemotherapy. Minimal residual disease (MRD) refers to the presence of these surviving cells that can be detected by flow cytometry or by PCR for informative genetic markers (1–3). The challenge is to eliminate the relapse-initiating ALL cells, and novel approaches specifically targeting residual leukemia cells are warranted (4–6).

Hematopoietic stem cells (HSCs) are located in the osteoblastic and the perivascular niches of the bone marrow (BM) (7, 8). Adhesion of leukemia cells to the BM has been found to contribute to chemoresistance of residual leukemia cells (6, 9–11). Integrins, a family of glycoprotein cell surface receptors composed of two subunits, alpha and beta (12), are responsible for cell adhesion to the extracellular matrix (ECM). The beta1 integrins are also known as very-late-activation antigens (VLAs). The integrin alpha4-chain (alpha4, also known as CD49d) non-covalently associates with the beta1 integrin chain, CD29, to form very-late-antigen-4 (VLA-4). VLA-4 is also referred as alpha4/beta1 or CD49d/CD29 and although there is an alternative beta-partner for alpha4, beta7, to form MadCAM, CD49d/CD29 is referred to throughout this manuscript as “the alpha4 integrin.” The alpha4 integrin binds to its counter receptors, including vascular cell adhesion molecule-1 (VCAM-1), fibronectin, or osteopontin (OPN) (13), and regulates retention and mobilization as well as to some degree cell cycle activity of immature progenitors in the BM (14). Alpha4 is expressed, among many other blood cells, on pre-B ALL cells (15, 16) and was recently quantified by real-time polymerase chain reaction in leukemia cells from 56 patients with relapsed ALL enrolled in the ALL-REZ BFM 2002 trial of the Berlin–Frankfurt–Münster study group (17). High alpha4 integrin mRNA expression was identified as an adverse risk factor in childhood ALL at first relapse. Therefore, we review here integrin alpha4 as a therapeutic target in drug resistance of leukemia.

## Integrin Structure

**Figure 1:**
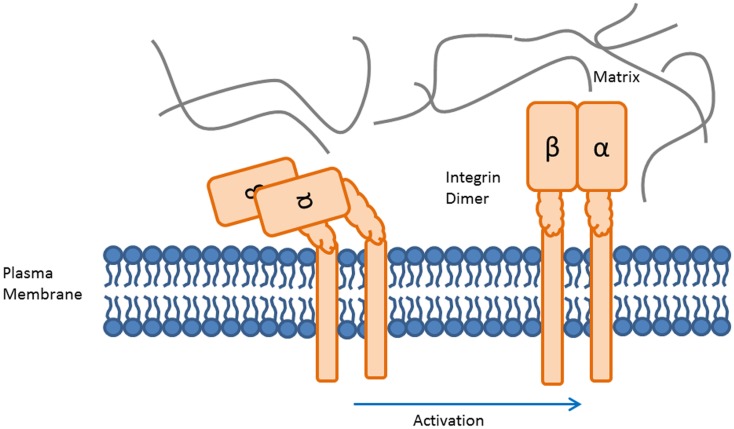
**Activation of the integrin heterodimer induces a conformational change**. The conformational states of the integrin heterodimer determine whether it functions for cellular adhesion or migration. The bent, inactive form of the integrin heterodimer prevents binding of ligands to the recognition region (left). Activation induced conformational change results in availability of the ligand binding region (right).

Integrins are a family of 24 heterodimeric cell surface proteins sharing significant structural and functional commonalities (13, 18, 19). They consist of an alpha- and a beta-chain, which are non-covalently bound. The alpha-chain predominantly defines the ligand specificity of the integrin. Conformational changes induced by approximation of the transmembrane regions of the integrin heterodimer open the ligand binding pocket. Ligand binding further affects integrin conformation, stretching the heterodimer from a bent to an extended conformation, shown in Figure 1 (20). The high-affinity form of integrins is stabilized by insertion of certain proteins from a multi-protein complex associated with the intracellular domain of integrins, e.g., talin (21). In the active conformation, integrins elicit intracellular (outside-in) signals through a variety of cell-type-dependent pathways, which include survival/apoptosis, cell cycle, metabolism, among others (12, 22–29). Integrins thus mediate physical adhesion to suitable matrices/ligands while at the same time providing outside-in signals regulating cell fate.

## Integrin Ligands

Integrin alpha4 binds to VCAM-1, a cell surface protein expressed on activated vascular endothelium and a host of other cells, as well as several ECM proteins, including OPN and the highly abundant fibronectin. VCAM-1 is a type I membrane protein expressed mainly on the surface of vascular endothelial cells throughout the vascular tree, but also on hematopoietic cells, although its function on these cells remains elusive. VCAM-1 expression on blood vessels is induced after cytokine stimulation, which is regulated either by increasing VCAM1 mRNA expression or mRNA stabilization (30). An alternative receptor for VCAM-1 to alpha4 is MadCAM, the alpha4/beta7 integrin heterodimer (31).

Fibronectin, a large glycoprotein dimer, binds primarily other matrix proteins, such as collagens and heparan sulfates, but contains moieties for integrin alpha4/beta1 binding (within the V-domain) as well as for alpha5 and the platelet integrin alphaV/beta3 (the RGD domain) (32). Experiments with molecules antagonizing fibronectin versus VCAM-1 binding, however, did not detect apparent effects of fibronectin-blockade on the trafficking of immature hematopoietic cells, whereas VCAM-1 inhibition interfered quantitatively with the interaction between hematopoietic stem/progenitor cells and BM (33). Matsunaga et al. have reported a fibronectin peptide based blockade of acute myeloid leukemia (AML) (34). As to the relevant ligand for alpha4 integrin in the stroma niche, contradictory data have been published. Specifically, Cradock and colleagues identified VCAM1 as the relevant ligand for normal hematopoietic cells, while the CS1 motif was apparently redundant (33). By contrast, Matsunaga et al. proposed adhesion to fibronectin as the molecular mechanism driving alpha4 integrin-mediated chemoresistance (35). If this was a general rule, then targeting the VLA-4-fibronectin interface would represent an even more leukemia cell specific target than VLA-4 proper. However, our own data show contributory roles of both ligands for chemoresistance of leukemia cells (and most strongly for VCAM1) at least *in vitro* (36), so that the situation currently remains unresolved.

Osteopontin is a negatively charged ECM glycoprotein, and has been described as a ligand for alpha4 integrin (37, 38), but alternative receptors include the alpha9 integrins (39). A role for OPN as a negative regulator of HSC proliferation and a mediator of HSC localization within BM has been proposed (40, 41). Which one of these alpha4 ligands is most critical for leukemia cell attachment-mediated drug resistance remains elusive.

## Integrin Intracellular Signaling

**Figure 2:**
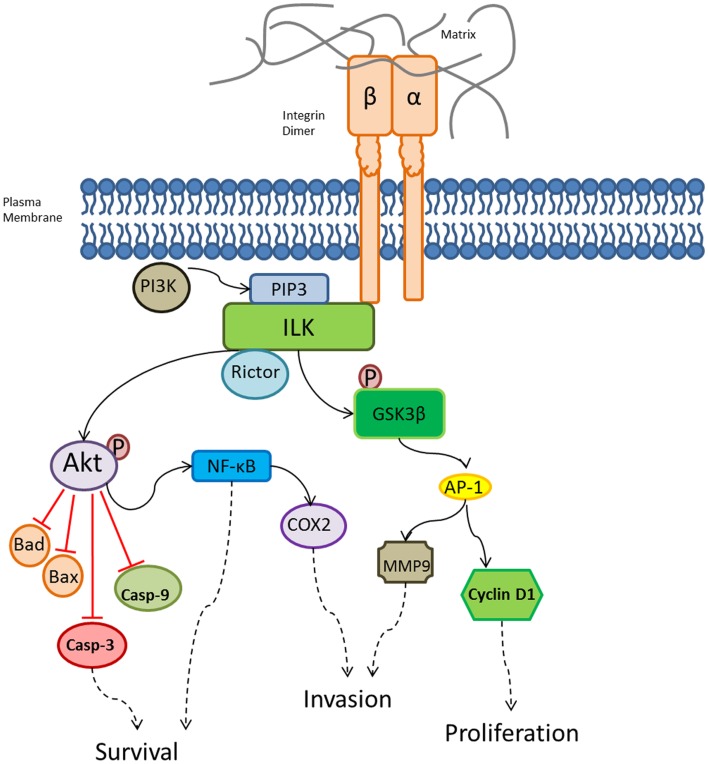
**Integrin intracellular signaling pathways regulated by ILK**. A variety of biological processes are regulated by ILK, which is a central player in multiple signaling cascades crucial for tissue homeostasis. ILK activation results in downstream effects responsible for survival, invasion, and proliferation. AP-1, activator protein 1; casp, caspase; GSK β, glycogen synthase kinase-3β; MMP9, matrix metalloprotease 9; NF-κB, nuclear factor-κB; P, phosphate; PI3K, phosphatidylinositol 3-kinase; PIP3, PtdIns(3,4,5)P3. Solid black arrows indicate activation, dashed black arrows indicate downstream effects, and the red lines indicate inhibitory effects.

Integrins can elicit intracellular signaling both directly and indirectly through other receptors (42). These are complex signaling mechanisms, which are briefly summarized here: Indirect intracellular signaling involves integrins forming complexes with receptor tyrosine kinase (RTK), which then interferes with activation of RTK by its normal ligand (43). A main structural and signaling protein involved in direct integrin signaling is integrin-linked kinase (ILK), which binds integrins (Figure 2). ILK forms multi-protein complexes with several key components involved with the cytoskeletal dynamics and intracellular signaling cascades. ILK kinase activity is dependent on PI3K and requires binding of PtdIns(3,4,5)P3 (PIP3) (44–46). Key players in cellular signaling that bind ILK specifically at the kinase domain include: PDK1, Akt, Rictor, Src. Rictor directly interacts with ILK, leading to the phosphorylation of Akt at serine 473 (26). This regulates cellular survival via caspase activation and nuclear factor-κB (NF-κB) stimulation (44, 47). ILK phosphorylates glycogen synthase kinase-3β (GSK3β) through phosphorylation on serine 9, resulting in the activation of activator protein 1 (AP-1), which then stimulates cyclin D1 and matrix metalloprotease 9 (MMP9) (44, 45, 48). Tabe et al. showed that ILK/Akt is a signaling pathway critical for survival of leukemic cells (49). Specifically, they demonstrated in a co-culture system of leukemic NB4 cells with BM-derived stromal mesenchymal stem cells (MSC), activation of ILK/Akt, extracellular signal-regulated kinase 1/2 (ERK1/2), signal transducers and activators of transcription 3 (STAT3), as well as Notch1/Hes. Blockade of PI3K or ILK signaling with pharmacologic inhibitors, LY294002 or QLT0267, resulted in induction of apoptosis in both leukemic cell lines and in primary AML samples. Muranyi et al. showed that targeting ILK and FMS-like tyrosine kinase-3 (FLT3) with an inhibitor of ILK and FLT3, OLT0267, is cytotoxic to AML stem cells using a long-term suspension culture system and a NOD/SCID mouse leukemia-initiating assay (50).

**Figure 3:**
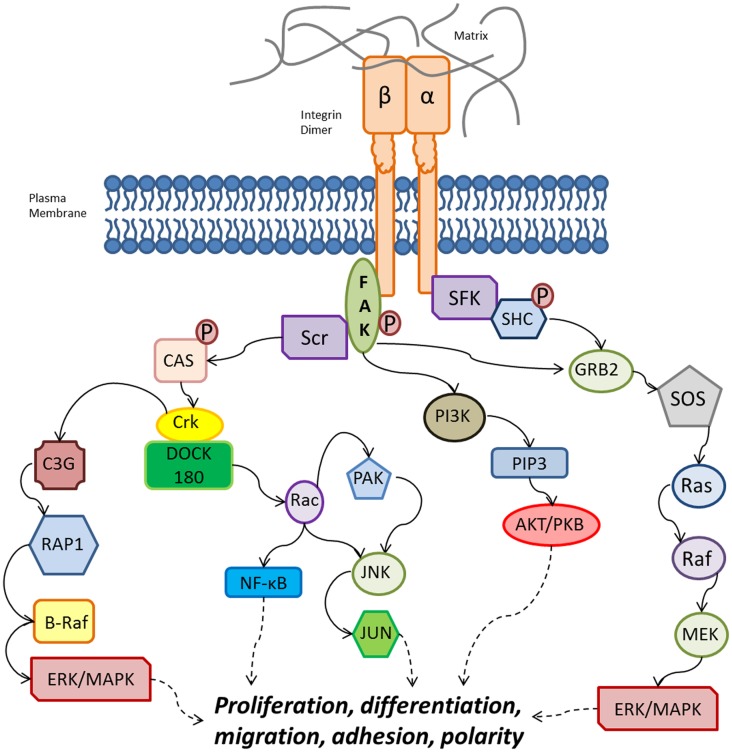
**Overview of integrin intracellular signaling cascades from both the alpha and beta subunits, leading to the activation of various cellular functions**. Binding of an alpha/beta integrin to the extracellular matrix ligands leads to activation of FAK. Note that other signaling pathways are stimulated by integrin heterodimers, but are not included for clarity and conciseness. FAK: focal adhesion kinase; GRB2, growth-factor-receptor-bound-2; P, phosphate group; PAK, p21-activated kinase; PI3K, phosphatidylinositol 3-kinase; PIP3, PtdIns(3,4,5)P3; PKB, protein kinase B; SFKs, Src-family kinases; SOS, son-of-sevenless. Solid black arrows indicate activation, dashed black arrows indicate downstream effects, and the red lines indicate inhibitory effects.

Direct intracellular signaling involves direct activation of tyrosine kinases by integrins. It has been described that integrin clustering activates tyrosine phosphorylation via focal adhesion kinase (FAK) (51). Integrin intracellular signaling involved the recruitment and activation of Src-family kinases (SFKs), which recruit FAK through the beta subunit (Figure 3). FAK can activate signaling from phosphatidylinositol 3-kinase (PI3K) to AKT/protein kinase B (PKB) through phosphatidylinositol-3,4,5-trisphosphate [PtdIns(3,4,5)P3], as well as recruiting Src to focal adhesions. Src can then phosphorylate CAS and paxillin, which further recruits the Crk–DOCK180 complex that results in the activation of Rac (52). The activation of Rac further activates p21-activated kinase (PAK), Jun amino-terminal kinase (JNK), and NF-κB (52–54).

Focal adhesion kinase can activate extracellular signal-regulated kinase (ERK)/mitogen-activated protein kinase (MAPK) via two pathways. First FAK can recruit C3G and RAP1 via Crk (55), which induces B-Raf activity and ERK/MAPK activation (12). The second pathway involves the growth-factor-receptor-bound-2 (GRB2) and son-of-sevenless (SOS) complex, which activates Ras–ERK/MAPK. The alpha subunit is also able to activate ERK/MAPK via SFK coupling, which phosphorylates the SHC, activating the GRB2–SOS complex and ERK/MAPK signaling downstream of Ras (56, 57).

The extracellular domain of alpha4 integrins mediates cell adhesion, while the cytoplasmic domain couples signaling and linkage with the cytoskeleton (13). The cytoplasmic tail of alpha4 integrin has been reported to bind to the signaling adaptor paxillin (58). The tight association of paxillin with the alpha4 tail leads to distinct biochemical and biological responses to integrin-mediated cell adhesion (58).

Lim et al. have reported that localized cAMP-dependent protein kinase (PKA) activation in pseudopodia of migrating cells phosphorylates alpha4 integrins to provide spatial cues governing cell motility. Specifically, they have shown that the alpha4 cytoplasmic domain is a Type I PKA-specific a-kinase anchoring proteins (AKAP) (59).

Rivera Rosado et al. have shown that cytoplasmic alpha4 tail associates with non-muscle myosin IIA (MIIA) independent of paxillin binding indicating a new mechanism for linking integrins to the actomyosin cytoskeleton and for regulating cell migration by integrins (60).

The interactions outlined above may regulate alpha4-specific adhesion in leukocytes, and it is conceivable, although it remains to be seen, that they may impact MRD.

In addition, Liu et al. have shown that chemoprotection of Jurkat T-ALL cells is integrin alpha4- or alpha5-mediated (61). The cytoplasmic domains of alpha-integrins have few sequences in common with the exception of the highly conserved membrane-proximal KXGFFKR motif (62). Interesting, Liu et al. have shown that reconstituted expression of alpha4δ, a truncated alpha4 integrin with KxGFFKR as cytoplasmic motif, in alpha4-deficient cells promoted chemoresistance to doxorubicin independent of alpha4-mediated adhesion of T-ALL cells (61).

Taken together, these observations indicate that the chemoprotective effects of leukemia cells associated with alpha4 integrin are at least in part mediated through integrin signaling and not through alpha4-mediated adhesion alone, although contributory roles of adhesion cannot be ruled out.

## Integrin-Bone Marrow Stromal Interactions Mediate Survival and Resistance of Leukemia Cells to Chemotherapy

As the BM is the most frequent relapse site for ALL (63), the BM has been considered a protective niche for leukemia cells (64). Previous studies have shown that ALL cell adhesion is mediated by alpha4 (65) and also that AML cell adhesion is specifically mediated by beta1 integrin, which leads to cell adhesion-mediated drug resistance (CAM-DR) (66). Mudry et al. conducted an *in vitro* investigation of one T-ALL and three pre-B ALL cell lines either in co-culture with stroma cells or on proteins isolated from the stromal matrix (11). When ALL cells were able to make direct contact with the stromal cells, the co-culture system maintained leukemia cell proliferation despite the presence of the chemotherapeutic agents, cytarabine or etoposide. This pro-survival effect was mediated through VCAM-1. Chemotherapeutic protection was not observed when leukemia cells were cultured in suspension above the stroma or on fibronectin alone. An interesting function of integrins beyond simple physical adherence is the activation of “outside-in” signals upon binding to their extracellular ligands, which apparently contribute to the integrin-mediated chemoprotective effects. Some of these chemoprotective signaling changes have been described below, as well as in Figures 2 and 3.

Astier and colleagues observed the inhibition of capase-3 and -7 activation by stimulation of β1 integrin in pre-B ALL cells. In the co-culture of BM stroma with pre-B ALL cells during chemotherapeutic treatment, the expression of the pro-apoptotic 23 kDa Bcl-2 protein was reduced (67). Wang et al. reported reduced levels of both PARP and cleaved Bcl-2 in ALL cells post-chemotherapy treatment, which was shown to be Akt-mediated (68). Previous studies by Fortney et al. showed that caspase 3 activity can be induced in ALL cells after treatment with the chemotherapeutic compounds, cytarabine or etoposide (69). Interestingly, this apoptotic effect is prevented in ALL cells co-cultured with human BM stroma cells.

The PI3K/Akt pathway (Figure 3) has been implicated in stroma cell-mediated chemoprotection and survival of leukemia cells (68). Additionally, gene expression analysis of leukemia cells with high versus low alpha4 expression identified 27 differentially expressed genes involved in ephrin, Rho GTPase, and PI3K/Akt pathways (17). More recently, it has been implicated that B-ALL cells interact with the BM-derived mesenchymal stromal cells through Notch-3 and -4 signaling (70). In a study by Bertrand et al., the inhibition of MEK with either mTOR or PI3K in the B-ALL cell line BLIN-2 resulted in rapid apoptosis of the leukemic cells (71).

Pillozzi et al. reported similar data in a co-culture system of ALL cells and BM mesenchymal stroma cells where hERG1 (human ether-à-go-go-related gene 1) channels have a role in the survival of leukemia cells via a signaling complex also containing beta1 integrin and CXCR4 (72). In addition, the PI3K/Akt pathway has been linked to regulation of drug transporting pumps, which may contribute to drug resistance (73): Ma showed high expression level of ST6GAL (β-galactoside α2, 6-sialyltransferase)-1 gene in leukemia cells. ST6GAL1 modulated the activity of (PI3K)/Akt signaling and regulated the expression of P-glycoprotein (P-gp) and multidrug resistance related protein 1 (MRP1) in leukemia cell lines. These data indicate that changes in P-gp based drug efflux mechanisms downstream of integrin signaling may contribute to chemoresistance. However, others have reported that MRP1, but not P-gp expression is under the control of the PI3K/Akt axis in AML blasts (74), so that definitive conclusions cannot be drawn at this point.

Recently, Andreeff and colleagues provided evidence of stroma-induced and alpha4-mediated NF-κB signaling in leukemia cells (75): Jacamo et al. have shown that VCAM1/VLA-4 activates NF-κB activation in leukemia and BM stromal cells and this crosstalk contributes to chemoresistance of leukemia cells (75).

Taken together, these studies demonstrate that integrin-BM stromal interactions trigger intracellular signaling changes, which then mediate chemoprotection. Interruption of this chemoprotective interaction-induced signaling would potentially overcome drug resistance. These findings indicate also that the effect of targeting integrin alpha4 goes beyond physical dislodgement of leukemia cells from the BM niche.

## Targeting of Alpha4 Integrin

Pre-clinical and clinical testing of new therapies against various integrins involved in different diseases is a rapidly emerging field (76–81). Over 260 anti-integrin drugs are currently in clinical evaluation, but only a few have been approved for clinical use. One of these is the anti-functional antibody, natalizumab (82), which targets both alpha4/beta1 and alpha4/beta7 (Table 1). Natalizumab, which is almost fully humanized, has been available in the clinic for treatment of relapsing–remitting multiple sclerosis as well as of complicated refractory inflammatory bowel disease with very high efficaciousness and for the most part good safety and tolerability. Long-term treatment has been associated in some patients with induction of neutralizing antibodies against the few remaining murine amino acid residues of the antibody, a scenario presenting as mild infusion-associated serum sickness and loss of efficacy (83). Infrequently, JC-virus-associated progressive multifocal leukoencephalopathy (PML) has been described after prolonged use, a complication, which is associated with a poor prognosis despite aggressive plasmapheresis (84). Long-term effects on hematopoiesis have been studied and did not provide any evidence for hematopoietic exhaustion in agreement with specific evidence in mice deficient for alpha4 integrin on hematopoietic cells (85, 86). We have evaluated the use of natalizumab in a xenograft model of primary leukemia and observed prolonged survival of mice treated with the combination of vincristine, dexamethasone, and l-asparaginase plus natalizumab (36). These data indicate the promise of this clinically approved antibody for ALL treatment. Another humanized antibody which is shown to inhibit alpha4beta7-mediated cellular adhesion is MLN-02 (87), which is a selective antagonist of alpha4/beta7 currently in clinical evaluation against Crohn’s disease and Ulcerative Colitis (88–90).

**Table 1:** **Summary of integrin alpha4 targeting drugs**.

Drug	Target	Disease	Reference	Drug class	Mechanism/ligand
Natalizumab	Alpha4beta1 and 7	MS; leukemia	(71)	Human monoclonal antibody	Non-competitive antagonism, VCAM
AJM300/HCA2969	Alpha4beta1 and 7	IBD, UC, Crohn’s	(81)	Orally available small molecule *N*-acetyl phenylalanine	Selective antagonist, VCAM
SB683699/firategrast	Alpha4beta1 and 7	IBD, MS, RA, asthma, Crohn’s	(82)	Orally available small molecule *N*-acetyl phenylalanine	Selective antagonist, VCAM
R-411/valategrast	Alpha4beta1 and 7	Asthma, arthritis	(83, 84)	Small molecule *N*-acetyl phenylalanine	Inhibit binding of alpha4 with receptors
IVL745	Alpha4beta1 and 7	Asthma	(90)	Small molecule inhalant	LDV, VCAM, and fibronectin
CDP323	Alpha4beta1 and 7	MS	(85, 86)	Orally available small molecule *N*-acetyl phenylalanine	Antagonist
THI0019	Alpha4beta1 and 7; alpha5beta1, alphaLbeta2	N/A	(91)	Small molecule	Agonist, binding at subunit interface
TBC3486	Alpha4beta1	N/A	(92)	Urea-based small molecule	Ligand mimetic Selective antagonist, VCAM-1
Bio-1211	Alpha4beta1 and 7	Asthma	(88)	Urea-based small molecule	Selective inhibitor LDV, fibronectin
Bio5192	Alpha4beta1	EAE, HSC mobilization	(78, 89)	Urea-based small molecule	High-affinity due to slow dissociation rate LDV
LLP2A	Alpha4beta1	Airway inflammation	(97, 98)	Peptidomimetic compound	Binds Trp188 and Gly190, close to binding sites for VCAM-1 and fibronectin
HMR-1031	Alpha4beta1	Asthma	(99, 100)	Small molecule inhalant	Selective antagonist VCAM-1 and fibronectin
Compound 7n	Alpha4	Asthma	(101)	Orally available small molecule	
MLN-02	Alpha4beta7	IBD	(74–77)	Humanized antibody	Selective antagonist, fibronectin

*MS, multiple sclerosis; IBD, inflammatory bowel disease; UC, ulcerative colitis; RA, rheumatoid arthritis; EAE, experimental autoimmune encephalomyelitis; LDV, leucine–aspartic acid–valine*.

Alternative molecules targeting alpha4 are under evaluation for a variety of diseases and have been summarized in more detail (78, 79, 91–93) (Table 1), although none are approved so far for clinical use in leukemia. There are two main classes of alpha4 integrin antagonists: urea-based and phenylalanine-based antagonists. The *N*-acetyl phenylalanine group consists of the small molecules AJM300 (94), SB683699 (Firategrast) (95), R-411 (Valategrast) (96, 97), and CDP323 (98, 99). These phenylalanine derivatives are novel compounds with improved potency against alpha4 integrins, through the formation of a cyclic peptide (100).

The urea-based antagonists are Bio-1211 (101), Bio5192 (92, 102), IVL745 (103), and TBC3486 (104), which is the parent molecule to THI0019 (105). These small molecule antagonists can further be categorized by the presence of a leucine–aspartic acid–valine (LDV) motif. The LDV motif contains a crucial aspartate recognized by integrin alpha4 (106–108). Alpha4 integrin has three LDV motifs in the extracellular sequence. The active-site motifs of integrin ligands can be reproduced synthetically as small molecules to provide both information regarding receptor–ligand binding and the development of therapeutic agents. For example, R-411 (valategrast), is metabolized to its active form RO0270608, which immediately reversed the binding of leukocytes to VCAM-1 (96). In early clinical trials, this molecule has shown encouraging results regarding pharmacokinetics and toxicity in patients with asthma (96, 97, 109). Of the LDV antagonists, Bio5192 is the only selective antagonists specifically for alpha4/beta1, while the others are dual antagonists. Another antagonist discussed here is a novel peptidomimetic compound that is specific for the biding site on alpha4/beta1 integrin heterodimer called LLP2A (110). LLP2A is a high-affinity ligand due to the replacement of the LDV motif with unnatural, modified amino acids (111). HMR 1031 is a selective VLA-4 receptor antagonist that blocks the binding of VCAM1 and fibronectin (112), but has shown poor efficacy in subjects with clinically persistent asthma (113). Compound 7n is a zwitterionic compound specifically modified from the VLA-4 antagonist compound 3, which is a 2-(phenylamino)benzoxazole derivative to improve oral bioavailability, which showed favorable efficacy in a mouse model of asthma (114). It remains to be seen if these novel alpha4 integrin inhibitors will be approved for clinical use.

## Conclusion

Integrins have been implicated in adhesion-mediated drug resistance of leukemic cells. As integrin alpha4 has been described in particular to be highly expressed in ALL (58, 102), this review focused on summarizing recent studies of integrin alpha4-associated cell survival and how to interrupt this chemoprotective binding. We have shown that integrin alpha4 is a promising target in the therapy against ALL (102). Interference with alpha4 not only deadheres leukemia cells physically from the chemoprotective BM niche, but may interrupt intracellular signaling changes critical for survival and resistance of leukemia cells. Several ways to target integrin alpha4 are under pre-clinical evaluation. Whether targeting of alpha4 integrin should be a strategy to prevent relapse, or whether it should be a treatment of relapsed ALL remains to be determined. Currently, the only clinically available anti-integrin alpha4 antagonist is natalizumab. Pre-clinical evidence has been provided that interference with alpha4-mediated adhesion of ALL cells can sensitize them to chemotherapy and thus facilitate eradication of ALL cells in an MRD setting (36). Finally, it remains to be determined whether toxicity of standard-risk ALL treatment can be decreased by combining administration of anti-alpha4 inhibitions with a reduced-dose chemotherapy.

## Conflict of Interest Statement

The authors declare that the research was conducted in the absence of any commercial or financial relationships that could be construed as a potential conflict of interest.
